# Crocin attenuates endoplasmic reticulum stress in methylglyoxal-induced diabetic nephropathy in male mice: MicroRNAs alterations and glyoxalase 1-Nrf2 signaling pathways

**DOI:** 10.22038/IJBMS.2022.65824.14479

**Published:** 2022-11

**Authors:** Vahid Radmehr, Akram Ahangarpour, Seyyed Ali Mard, Layasadat Khorsandi

**Affiliations:** 1 Student Research Committee, Department of Physiology, Ahvaz Jundishapur University of Medical Sciences, Ahvaz, Iran; 2 Medical Basic Sciences Research Institute, Physiology research center, Department of Physiology, School of Medicine, Ahvaz Jundishapur University of Medical Sciences, Ahvaz, Iran; 3 Alimentary tract research center, Ahvaz Jundishapur University of Medical Sciences, Ahvaz, Iran; 4 Department of Anatomical Sciences, School of Medicine, Medical Basic Sciences Research Institute, Cellular, and Molecular Research Center, Ahvaz Jundishapur University of Medical Sciences, Ahvaz, Iran

**Keywords:** Crocin, Diabetic nephropathy, ER stress, Methylglyoxal, Nephropathy, Nrf2

## Abstract

**Objective(s):**

Accumulation of methylglyoxal (MGO) occurs in diabetes. MicroRNA-204 is an important intracellular marker in the diagnosis of endoplasmic reticulum stress. Crocin (Crn) has beneficial effects for diabetes, but the effect of Crn on MGO-induced diabetic nephropathy has not been investigated. The current research evaluated the effects of Crn and metformin (MET) on diabetic nephropathy induced by MGO in male mice.

**Materials and Methods:**

In this experimental study, 70 male NMRI mice were randomly divided into 7 groups: control, MGO (600 mg/Kg/d), MGO+Crn (15, 30, and 60 mg/kg/d), MGO+MET (150 mg/kg/d), and Crn60 (60 mg/kg/d). Methylglyoxal was gavaged for four weeks. After proving hyperglycemia, Cr and MET were administered orally in the last two weeks. Biochemical and antioxidant evaluations, microRNA expression, and histological evaluation were assessed.

**Results:**

The fasting blood glucose, urine albumin, blood urea nitrogen, plasma creatinine, malondialdehyde, Nrf2, miR-204, and miR-192 expression increased in the MGO group. These variables decreased in Crn-treated animals. The decreased levels of superoxide dismutase, catalase, glyoxalase 1, Glutathione, and miR-29a expression in the MGO group improved in the diabetic-treated mice. Histological alterations such as red blood cell accumulation, inflammation, glomerulus diameter changes, and proximal cell damage were also observed.

**Conclusion:**

Our study indicated that Crn and MET attenuated renal damage by inhibiting endoplasmic reticulum stress.

## Introduction

Diabetic nephropathy (DN) is a microvascular complication of type II diabetes. It is reported that 20 to 40% of people with diabetes suffer from DN (1). MGO is one of the most potent reactive dicarbonyl species generated by glucose, lipid, and amino acid metabolism that accumulates in diabetic people (2). Also, MGO as the major precursor of advanced glycation end products (AGEs) can induce oxidative stress (3,4), disrupt the function of β cells in diabetes (5), and play an important role in DN formation (6). MGO-induced oxidative stress is interrelated to endoplasmic reticulum (ER) stress through intracellular mediators (7). On the other side, MGO directly stimulates the production of ROS by binding to its receptors and causes ER stress (8). ER stress activates an intracellular signaling pathway called the unfolded protein response. This intracellular response can relieve ER stress by destroying unfolded proteins (9). Double-stranded RNA-activated protein kinase-like ER kinase (PERK) is one of the unfolded protein response sensors that reduce ER stress by activating downstream signaling pathways (10). A report confirms that ER stress causes damage to podocytes and leads to proteinuria in diabetic mice (11). Also, the accumulation of MGO-derived AGEs in the glomerulus contributes to the symptoms of diabetic nephropathies such as glomerular basement membrane thickening, mesangial expansion, and albuminuria (12). 

Glyoxalase 1 (GLO1) potently metabolizes MGO into a non-toxic metabolite with the help of glutathione (GSH) as a reducing cofactor (13). The renoprotective effect of GLO1 was revealed previously (14). The nuclear factor erythroid 2–related factor 2 (Nrf2) is involved in detoxification of MGO-induced glucotoxicity (15). Previously, the role of Nrf2 was investigated in the pathogenesis of DN (16). Nrf2 positively regulates GLO1 levels at high MGO conditions and protects renal cells during dicarbonyl stress (17). MicroRNAs (miRNAs) as biomarkers may predict the onset of DN. High levels of miR-204 in the kidney are crucial to protect against diabetic kidney damage (18). Furthermore, the profibrotic effect of miR-192 and the anti-fibrotic effect of miR-29a have been identified in rodent kidney disease (19,20). It is well known that Crn is one of the active components of Crocus sativus with antifibrotic, anti-inflammatory, and antioxidant properties (21). Also, it was reported that MET (as a positive control in our study) can control blood glucose levels by lowering hepatic glucose and has protective and antifibrotic effects against kidney damage (22). In recent years, inhibition of AGEs formation is one of the effective strategies to prevent DN. However, no experimental study has been conducted on the effect of Crn in reducing ER stress by examining ER stress-related microRNAs in diabetic kidneys. Focused on this, we investigate whether Crn diminished ER stress in MGO-induced DN.

## Materials and Methods

***Chemicals***

MGO and Crn were purchased from Sigma–Aldrich (St. Louis, MO, USA). Xylazine and Ketamine from Alfasan Co. (Netherlands), MET from Solar bio (South Korea), High Pure RNA Isolation Kit (Roche, Germany), and miScript II RT Kit (QIAGEN, GmbH, Germany) were purchased. Insulin level was measured using Monobind Inc, USA kit. Nrf2 level, antioxidant biomarkers, and GSH content were assessed by ZellBio GmbH, Germany kits. Also, fasting blood glucose (FBG) and renal function test were measured using colorimetric assay kits (Pars Azmoon, Co, Iran).

***Experimental design***

**Figure 1:**
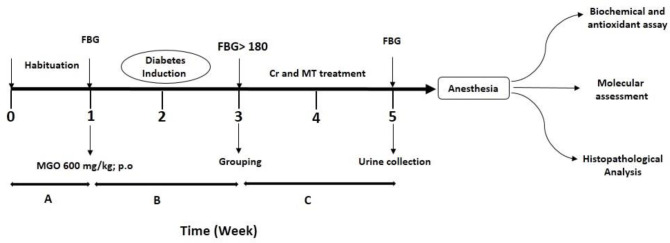
Diagrammatic representation of experimental protocol in male mice. A: Habituation lasted one week, B: Diabetes induction by MGO (600 mg/kg), C: Administration of Crn (15, 30, and 60 mg/kg) and metformin (150 mg/kg). At the end of 3^rd^ week, diabetic mice were selected based on their FBG levels and divided into MGO+Crn15: diabetic + Crocin 15 mg/kg, MGO+ Crn30: diabetic + Crocin 30 mg/kg, MGO+Crn60: diabetic + Crocin 60 mg/kg, MGO+ MET: diabetic + Metformin 150 mg/kg, Crn60: Crocin 60 mg/kg lasted 2 week

The experiment was performed for four weeks after one week of habituation of the animals to the laboratory environment (Figure 1). At the end of the experiment, urine was collected by keeping the animals in metabolic cages for 24 hr. Animals were fasted for 6 hr to measure FBG; then anesthetized with ketamine and xylazine to collect kidney tissues for biochemical evaluations and histological study. The blood samples collected from the heart were centrifuged at 3000 rpm for 15 min and the plasma samples were stored at -20 °C.

***Animals***

A total of 70 adult male NMRI mice (four weeks old) were obtained from Ahvaz Jundishapur University of Medical Sciences, Ahvaz, Iran; attributed to AJUMS practical animal care guidelines with an ethics committee grantee No. (IR.AJUMS.ABHC.REC.1397.076). The animals were routinely fed, the room temperature was 20 ± 4 °C, and a 12 hr light/12 hr dark cycle was set. Nephropathy was induced by MGO (600 mg/kg/day, gavage) administration (23) for 28 consecutive days. The animals were divided into seven groups of 10 mice :(1) Cont (Control), (2) MGO (600mg/kg/day, orally by gavage), (3) MGO + Crn15 (15 mg/ kg), (4) MGO + Crn30 (30 mg/kg), (5) MGO + Crn60 (60 mg/kg), (6) MGO + MET (150 mg/kg) (24), and (7) Crn60 (60 mg/kg) as an effective dose from pilot study. Crn and MET (for 14 days) were administered by gavage. On the fourteenth day, the FBG levels were measured from the tail vein after six hours of fasting. Then, animals with FBG above 180 mg/dl were considered diabetic and were selected for subsequent experiments.

***Experimental measurements***

The left kidney tissue was homogenized in Tris–HCl buffer, centrifuged for 15 min at 10,000 g, and supernatants were used to assess kidney Malondialdehyde (MDA) and enzymatic activity. The measuring of GLO1 activity was applied to assess MGO detoxification. The FBG, blood urea nitrogen (BUN), Creatinine (Cr), and albumin concentration were measured using an autoanalyzer (BT3000, Italy) and diagnostic assay kits (Pars Azmoon, Iran). Renal function was determined by calculating the creatinine clearance (mL/min) as glomerular filtration rate (GFR), according to the following standard formula:

GFR= Urine Cr × Urine volume / Plasma Cr (25).

At least six repeats were considered for each variable.

***MicroRNAs extraction and cDNA synthesis***

Total RNA was isolated from the frozen kidney tissue using the RNeasy Plus Mini kit. After detection of RNA purity and concentration, one microgram of the total RNA was used for cDNA synthesis by the miScript II RT Kit.

***Quantitative real-time PCR (qRT-PCR)***

The levels of MicroRNAs [miR-204 (MIMAT0000237), miR-29a (MIMAT0004631), or miR-192 (MIMAT0000517), and (QIAGEN)] were characterized using qRT-PCR according to the previous study (26).

***Histopathological analysis***

The kidney tissue samples were stained with H&E in the histology lab. Then, six microscopic images per animal were used to assess red blood cell (RBCs) accumulation, inflammation, glomerulus diameter, and proximal cell damage using a digital microscope (BMZ-04- DZ, Behin Pajouhesh ENG. CO., Iran). The mean of 10 fields was considered for each slide.

***Statistical analysis***

Results were represented as mean ± SEM using GraphPad Prism 9 for Windows (GraphPad Software, San Diego, CA, USA). One-way analysis of variance (ANOVA) was used for comparison between groups, followed by *post hoc* high significant difference (HSD) or chi-square test (with Bonferroni correction method). Also, Kruskal-Wallis non-parametric test followed by Dunn’s test was used to evaluate inflammation of inflammatory cells, RBCs congestion, and proximal cell damages, and the data were reported as median ± interquartile range (median ± IQR). Also, *P*<0.05 was considered significant.

## Results

***Effect of Crocin and metformin on FBG, kidney function, and biochemical indexes***

**Figure 2:**
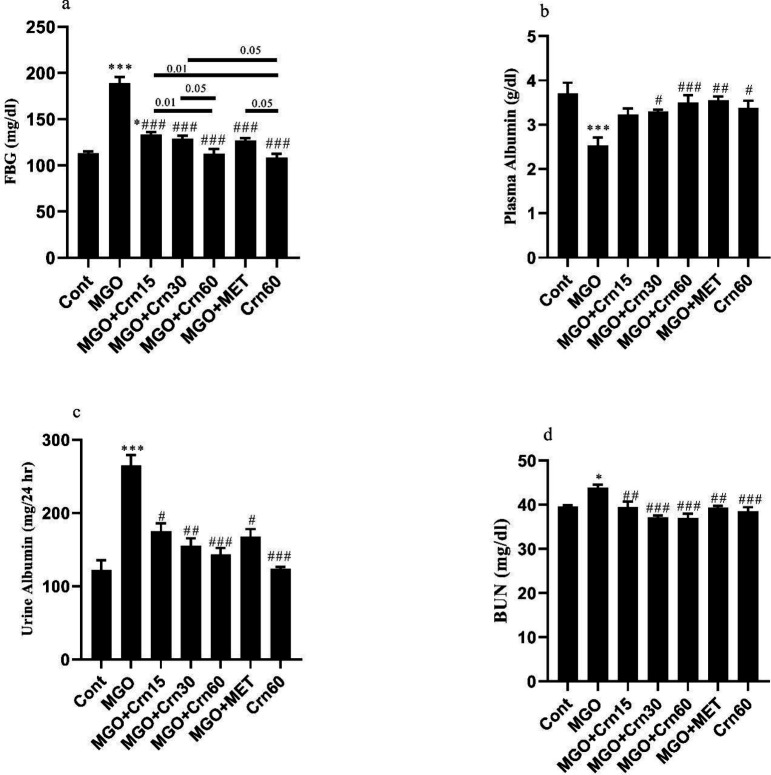
Effects of MGO, Crn, and MET on FBG a, Plasma albumin b, Urine albumin c, and BUN d in DN induced by MGO

Our finding indicated that the levels of FBG (*P*<0.001) increased in the MGO group compared with Cont (Figure 2). Treatment with Crn 30 (*P*<0.01), 60 (*P*<0.001), and MET (*P*<0.01) reduced the FBG level in diabetic mice. The high dose of crocin was more effective in lowering FBG levels than medium (*P*<0.05) and low doses (*P*<0.01). Also, Crn60 has a better function than MGO+Crn15 (*P*<0.001), MGO+Crn30 (*P*<0.01), and MGO+MET (*P*<0.01). Plasma level of albumin was reduced in the MGO group compared with Cont (*P*<0.05) and was increased in the MGO+Crn60 (*P*<0.05), MGO+MET (*P*<0.01), and Crn60 (*P*<0.05) groups compared with the MGO group. Urine albumin increased in the MGO (*P*<0.001), MGO+Crn15 (*P*<0.01), and MGO+MET (*P*<0.05) groups. Among the diabetic-treated groups, Crn 30 (*P*<0.05) and 60 (*P*<0.01) were able to reduce urine albumin. In addition, the difference between Crn60 and MGO+Crn15 groups was significant (*P*<0.05). BUN increased in the MGO group (*P*<0.05), while treatment with Crn and MET significantly reduced it. Also, our study showed that Crn60 had a significant difference with the MGO group (*P*<0.001) (Figure 2).

The Cr level of 24 hr urine decreased in diabetic mice when compared with Cont (*P*<0.001) (Table 1). This variable significantly increased in diabetic-treated mice except for the MGO+Crn15 group. Also, the difference between the MGO+Crn15 and Crn60 groups was significant (*P*<0.05). Urine volume increased in the MGO (*P*<0.001) and MGO+Crn15 (*P*<0.05) groups compared with Cont, but it was significantly decreased in Crn and MET-treated mice. Also, the urine volume in the Crn60 group was lower than the MGO+Crn15 (*P*<0.05). A significant increase was observed in plasma Cr level in the MGO group (*P*<0.01), while Crn and MET significantly reduced it close to Cont. Also, GFR decreased in the MGO group (*P*<0.001) and it was efficiently preserved close to the normal range in all diabetic-treated mice (Table 1). 

***Effect of crocin and metformin on lipid peroxidation, antioxidant enzymes, GSH, GLO1, and Nrf2 levels***

**Figure 3:**
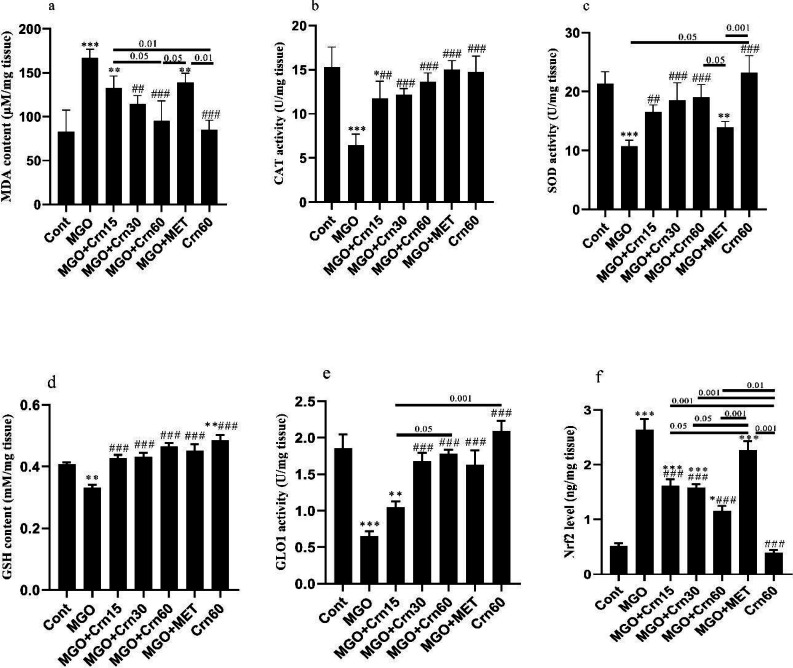
Effects o f MGO, Crn, and MET on the kidney levels of MDA a, CAT b, SOD c, GSH d, GLO1 e, Nrf2 f in DN induced by MGO in male mice

Our evaluations showed that the MDA levels as the best indicator of lipid peroxidation increased in the MGO (*P*<0.001) and MGO+Crn15 (*P*<0.01) group. It was decreased in MGO+Crn30 (*P*<0.01) and MGO+Cr60 (*P*<0.001) groups. The level of MDA in MGO+Crn60 was lower than in MGO+MET and MGO+Crn15 groups (*P*<0.05). Also, the MDA level in Crn60 was lower than MGO+Crn15 and MGO+MET groups (*P*<0.001). Significant reduction of catalase (CAT) level and superoxide dismutase (SOD) activity was observed in the MGO group (*P*<0.001). Treatment with Crn at all doses significantly improved the levels of these enzymes. Also, a significant reduction in SOD levels was observed in the MGO+MET compared with the MGO+Crn60 (*P*<0.05) and Crn60 (*P*<0.001) groups (Figure 3). The GSH content decreased in the MGO group (*P*<0.01). However, this variable increased in all diabetic-treated mice compared with the MGO group (*P*<0.001). Also, the GSH content in the Crn60 group was higher than Cont (*P*<0.01) and MGO groups (*P*<0.001). MGO significantly reduced GLO1 activity (*P*<0.001), while Crn 30, 60, and MET improved it (*P*<0.001). GLO1 activity in Crn60 (*P*<0.001) and MGO+Crn60 (*P*<0.05) groups was higher than MGO+Crn15 group. The Nrf2 level significantly increased in all MGO-treated mice compared with Cont, while Crn at all doses decreased it (*P*<0.001). Nrf2 levels in MET-treated mice were significantly higher than in Crn-treated mice. Also, the difference between Crn60 and other treated groups was significant (Figure 3).

***Effect of crocin and metformin on the kidney histology***

**Figure 4:**
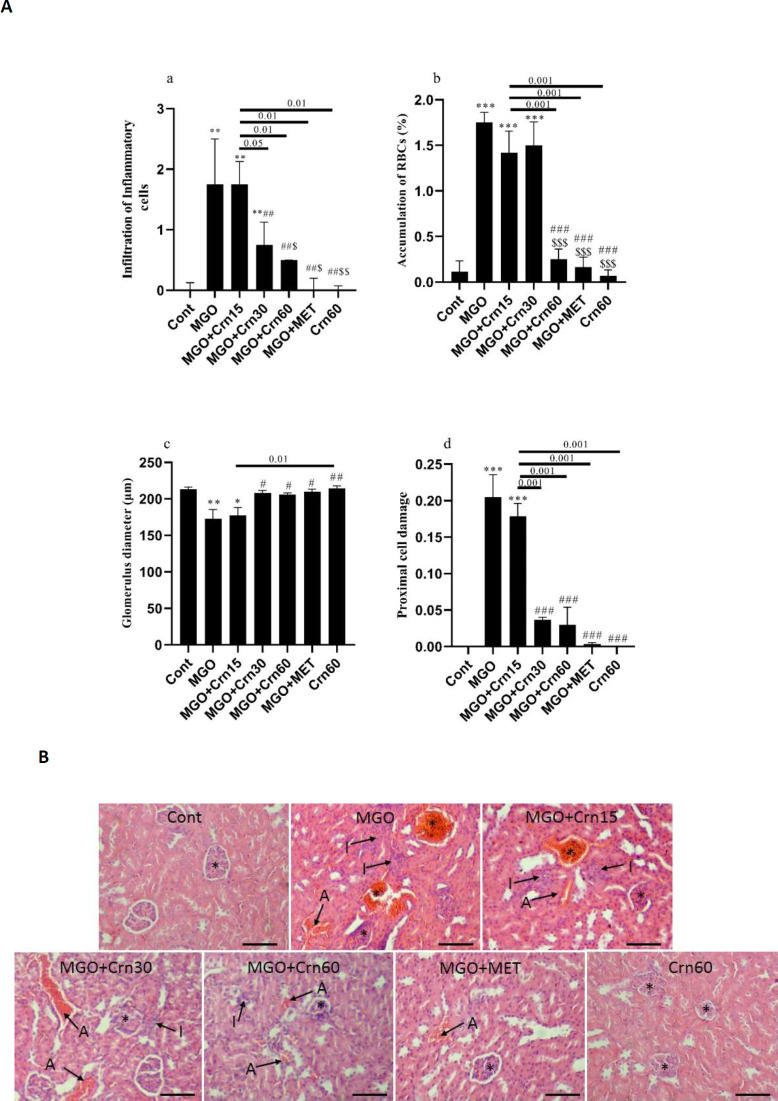
**a.** Effects of Crn and MET on the kidney morphology in male mice including inflammation a, RBCs accumulation b, Glomerulus diameter c, and proximal cell damage d in diabetic nephropathy induced by MGO

Renal histological examination showed that MGO caused many changes in renal morphology, including glomerular atrophy, proximal cell degeneration, inflammatory cell infiltration, RBC congestion, and tubular swelling compared with normal renal morphology. Infiltration of inflammatory cells increased in the MGO, MGO+Crn15, and MGO+Crn30 groups compared with Cont (*P*<0.01), and it was decreased in the MGO+Crn30, MGO+Crn60, and MGO+MET groups compared with the MGO group (*P*<0.01). Inflammation showed a remarkable decrease in a dose-dependent manner in the Crn-treated animals. The effect of Crn15 and Crn30 was significantly less than the other treatments. Treatment with Crn at a high dose and MET decreased the accumulation of RBCs (*P*<0.001). The levels of RBCs congestion in the Crn60 group were lower than in MGO, MGO+Crn15, and MGO+Crn30 groups (*P*<0.001). Also, MGO+Crn60 and MGO+MET remarkably decreased ABCs congestion compared with MGO+Crn30 (*P*<0.001). In addition, the diameter of the glomerulus significantly decreased in MGO and MGO+Crn15 groups compared with Cont, while treatment with Crn30, 60, and MET significantly improved it. Moreover, the difference between MGO+Crn15 and Crn60 groups was significant (*P*<0.001). Administration of Crn30, 60, and MET significantly decreased proximal cell damages compared with the MGO group (*P*<0.001). Further, the difference between the MGO+Crn15 group and other treated groups was significant (Figures 4a and 4b).

***Effect of Crocin and metformin on the kidney expression of miR-204, miR-192, and miR-29a***

**Figure 5:**
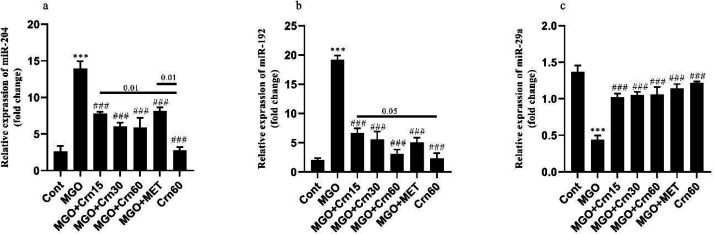
Effects of Crn and MET on miR-204 a, miR-192 b, and miR-29a c levels in DN induced by MGO in male mice

The results of the miRNAs expression showed that MGO increased the miR-204 and miR-192 expression (*P*<0.001), and decreased the miR-29a expression compared with Cont (*P*<0.01). However, administration of Crn 30 and 60 reversed the expression of miR-204 (*P*<0.01). The expression of miR-204 in the Crn60 group was lower than in MGO+Crn15 and MGO+MET groups (*P*<0.01). Also, MGO+Crn60 effectively decreased the expression of miR-192 (*P*<0.001). Furthermore, the expression of miR-192 in the Crn60 group was lower than in MGO+Crn15 (*P*<0.001). On the other hand, the expression of miR-29a increased in Crn and MET-treated animals compared with the MGO group (*P*<0.001). Additionally, Crn60 did not change miRNA levels when compared with Cont (Figure 5).

At the same time, the Control group received normal saline. Finally, after anesthesia, various factors were evaluated

Crn: Crocin; MGO: Methylglyoxal; MET: Metformin; FBG: Fasting blood glucose

Cont: Control; MGO: Diabetic with methylglyoxal administration; MGO + Crn15: Diabetic + Crn 15 mg/kg; MGO + Crn30: Diabetic + Crn 30 mg/kg; MGO + Crn60: Diabetic + Crn 60 mg/kg; MGO + MET: Diabetic + Metformin; Crn60: Crn 60 mg/kg

Data are presented as mean ± SEM, n=6 in each group. * Significant difference with Cont; # significant difference with MGO; 1 symbol *P*<0.05; 2 symbols *P*<0.01; 3 symbols *P*<0.001

Crn: Crocin; MGO: Methylglyoxal; MET: Metformin; FBG: Fasting blood glucose; BUN: Blood urea nitrogen; DN: Diabetic nephropathy

**Table 1:** Effect of crocin on Urine Cr, Urine Volume, Plasma Cr and glomerular filtration rate in male mice

**Groups**	**Urine Cr** **(mg/dl)**	**Urine volume** **(ml/24 hr)**	**Plasma Cr** **(mg/dl)**	**GFR** **(ml/min)**
**Cont**	35.25±1.03	0.64±0.014	0.26±0.007	86.22±2.15
**MGO**	26.25±1.03^***^	0.77±0.011 ^***^	0.36±0.008 ^**^	56.67±2.78 ^***^
**MGO+Crn15**	30±0.81 ^**^	0.70±0.006 ^*##^	0.26±0.004 ^#^	79±3.75 ^##^
**MGO+Crn30**	30.75±0.75 ^*#^	0.66±0.011 ^###^	0.26±0.008 ^##^	78.53±3.43 ^#^
**MGO+Crn60**	31±0.48 ^##^	0.64±0.007 ^###^	0.23±0.006 ^###^	82.94±2.22 ^##^
**MGO+MET**	32.50±0.64 ^###^	0.66±0.011 ^###^	0.25±0.004 ^##^	87.56±1.61 ^###^
**Crn60**	34.75±0.48 ^###^^$^	0.64±0.014 ^###^^$^	0.25±0.004 ^##^	90.98±4.22 ^###^

Cont: Control; MGO: Diabetic with Methylglyoxal administration; MGO + Crn15: Diabetic + Crn 15 mg/kg; MGO + Crn30: Diabetic + Crn 30 mg/kg; MGO + Crn60: Diabetic + Crn 60 mg/kg; MGO + MET: Diabetic + Metformin; Crn60: Crn 60 mg/kg

MGO: Methylglyoxal; Crn: Crocin; MET: Metformin

Data are presented as mean ± SEM, n = 10 in each group. *P-*values were calculated by analysis of variance (ANOVA) test (with Tukey's HSD as *post hoc *analysis) or chi-square test (with Bonferroni correction method). * Different from Cont; # different from MG, $ different from MG, $ different from MGO + Crn15; 1 symbol *P*< 0.05; 2 symbols *P*< 0.01; 3 symbols *P*<0.001

Cont: Control; MGO: Diabetic with Methylglyoxal administration; MGO + Crn15: Diabetic + Crn 15 mg/kg; MGO + Crn30: Diabetic + Crn 30 mg/kg; MGO + Crn60: Diabetic + Crn 60 mg/kg; MGO + MET: Diabetic + Metformin; Crn60: Crn 60 mg/kg

Data are presented as mean ± SEM, n=6 in each group. * Significant difference with Cont; # significant difference with MGO; 1 symbol *P*<0.05; 2 symbols *P*<0.01, 3 symbols *P*<0.001

MGO: Methylglyoxal; Crn: Crocin; MET: Metformin; MDA: Malondialdehyde; CAT:Catalase; SOD: Superoxide dismutase; GSH: Glutathione; GLO1: Glyoxalase 1

Cont: Control; MGO: Diabetic with Methylglyoxal administration; MGO + Crn15: Diabetic + Crn 15 mg/kg; MGO + Crn30: Diabetic + Crn 30 mg/kg; MGO + Crn60: Diabetic + Crn 60 mg/kg; MGO + MET: Diabetic + Metformin; Crn60: Crn 60 mg/kg

Data for graphs a, b, and d are presented as median ± IQR, and graph c is presented as mean ± SEM, n=6 in each group. * Significant difference with Cont; # significant difference with MGO; $ significant difference from MGO + Crn30; 1 symbols *P*<0.05; 2 symbols *P*<0.01; 3 symbols *P*<0.001

**b.** Effects of Crn and MET on the kidney histology in DN induced by MGO in male mice (hematoxylin & eosin staining) (I: inflammation, A: RBCs accumulation, Magnification: ×250, Scale bars: 200 µm, *: Glomeruli)

Cont: Control group showed normal structure in the glomerulus and proximal epithelial cells. MGO: diabetic with Methylglyoxal administration was severely damaged. Glomerulus atrophy, proximal epithelial cell swelling, accumulation of RBC, and inflammatory cells were seen

MGO: Diabetic with Methylglyoxal administration; MGO + Crn15: Diabetic + Crn 15 mg/kg; MGO + Crn30: Diabetic + Crn 30 mg/kg; MGO + Crn60: Diabetic + Crn 60 mg/kg; MGO + MET: Diabetic + Metformin; Crn60: Crn 60 mg/kg

Crn: Crocin; MET: Metformin; RBCs: Red blood cell; DN: Diabetic nephropathy; MGO: Methylglyoxal

Cont: Control; MGO: Diabetic with methylglyoxal administration; MGO + Crn15: Diabetic + Crn 15 mg/kg; MGO + Crn30: Diabetic + Crn 30 mg/kg; MGO + Crn60: Diabetic + Crn 60 mg/kg; MGO + MET: Diabetic + Metformin; Crn60: Crn 60 mg/kg

Data are presented as mean ± SEM, n=6 in each group. * Significant difference with Cont; # significant difference with MGO; 2 symbols *P*<0.01; 3 symbols *P*<0.001

Crn: Crocin; MET: Metformin; DN: Diabetic nephropathy; MGO: Methylglyoxal

## Discussion

Diabetic microvascular complications such as DN can be caused by methylglyoxal. In recent years, plant-derived chemical compounds have been widely used to inhibit AGEs formation (27). 

Several studies indicated that MGO could induce hyperglycemia, which contributes to AGEs production in the kidney. Increased production and insufficient removal of MGO are responsible for AGEs accumulation, which is related to albuminuria and mesangial expansion (28). Our results showed that oral administration of MGO caused albuminuria; however, treatment with Crn improved it. An increase in BUN level is one of the glomerular damage signs. In fact, the elevation of BUN, plasma Cr, and reduction of plasma albumin occurs in nephropathy. The current study showed that Crn could ameliorate BUN, plasma Cr, and albumin levels by improving filtration function through its antioxidant property. GFR is the best measure of kidney function, which gradually declines by renal damage. In this study, Cr clearance used for estimation of GFR indicated a significant reduction in the MGO group, while treatment with Crn improved it.

Accumulation of intracellular MGO leads to both oxidative stress and ER stress. It was reported that MGO treatment increases MDA levels and decreases SOD and CAT activity in kidney cells (29). In addition, MGO causes structural changes, including glomerular basement thickening and mesangial expansion in the rat kidneys (12). As a safe and natural compound, antioxidants can reduce MGO content in the kidney (30). Hence, our results demonstrated that MGO induced oxidative stress and elevated lipid peroxidation in mice kidneys through increased MDA levels and decreased antioxidant enzymes. The current result is in agreement with our previous study, in which hyperglycemia increased MDA and decreased SOD and CAT (31). Also, we found a reduction in MDA level and an enhancement in GSH, SOD, and CAT activity in the kidney of Crn-treated mice. It was clearly shown that safranal therapy effectively inhibits oxidative stress (32). As observed, Crn protected kidney cells against oxidative damage through its antioxidant property. 

During excessive glycolysis, GLO1 dysfunction leads to dicarbonyl stress (33). A previous study revealed that overexpression of GLO1 could suppress oxidative damage in diabetic rats and improve the pathological features of diabetic complications, such as DN (13). Regarding these reports, we found that GLO1 activity was reduced in the MGO group, and Crn exhibited ameliorative effects via enhancement of the GLO1 function. Transcription factor Nrf2 regulates gene expression of many antioxidant enzymes and phase II detoxification enzymes such as GLO1. There are different reports about the Nrf-2/GLO1 signaling pathway. It has been shown that increased expression of Nrf2 and GLO1 improves diabetes-induced renal glucotoxicity through MGO detoxification (34). Another previous study showed that MGO did not affect the Nrf2 mRNA level and reduced GLO1 mRNA level in the liver of MGO-diabetic rats (23). Also, it has been reported that MGO decreased GlO1 activity and Nrf2 levels in the kidney of MGO-diabetic mice (34). The findings of a study determined that MGO decreased the Nrf2 protein expression in mouse glomerular mesangial cells (35). This finding is in contrast to our study in which MGO increased the Nrf2 level. Another study reported that treatment with MGO decreased GLO1 activity (36). Our results showed that MGO reduced GLO1 activity which agrees with the present findings. However, treatment with Crn reversed the adverse effects of MGO on GLO1 activity and Nrf2 levels in the kidney of MGO-treated mice. Our results showed that Crn increased GLO1 activity and GSH content. The changes in GSH activity reflect the degree of oxidative injury (37), indicating that the induction of GLO1 after Crn treatment may contribute to the nephroprotective properties of Crn. Histopathological evaluations confirmed the study’s results mentioned above, indicating the beneficial effects of Crn.

Finally, our study was completed by examining changes in miRNA levels. For this purpose, we evaluated the levels of miR-204, miR-192, and miR-29a alterations in the kidney of the diabetic model induced by MGO. MicroRNAs can precisely identify signaling pathways contributing to the pathogenesis of DN (20). Previously, the role of miR-204 in ER stress was identified (38). Another study indicated that miR-204 directly targets and inhibits PERK signaling (39). Results from quantitative RT-PCR showed that MGO increased miR-204 expression in renal tissue, and treatment with Crn reduced it. This finding exhibits that under high levels of MGO, overexpression of miR-204 following GLO1 deficiency can stimulate ER stress. In contrast, treatment with Crn improves GLO1 function, reduces MGO levels, and modulates microRNA expression. In agreement with our results, a study showed that accumulation of ROS leads to ER stress that can be detected by miR-204 overexpression (40). Previous studies suggest that overexpression of miR-204 indirectly alters the Nrf2 levels (26). Other studies indicated a direct relation between PERK and Nrf2. Generally, PERK keeps cell survival by activating Nrf2 and causes an essential cytoprotective effect against ER stress. In other words, at the beginning of ER stress, the activation of Nrf2 along with the helpful effects of GSH inhibits the ER stress by maintaining cellular redox balance (41). So, it seems that the Nrf2 levels are affected by miR-204 expression. Therefore, the increased level of Nrf2 in the MGO group is confirmed by current reports.

Subsequently, the formation of fibrosis in the kidneys of MGO-treated mice was investigated through related miRNAs. Evaluation of the renal fibrosis-related miRNAs expression showed that MGO elevated miR-192 and reduced miR-29a expression, whereas Crn restored both of them. Similarly, Wang *et al*. (42) reported that the miR-29 family reduces renal fibrosis. These findings indicated that Crn exhibits an antifibrotic effect by decreasing the miR-192 expression and increasing the miR-29a expression. Also, the expression of miR-192 increases in renal fibrosis induced by high glucose treatment (43). It has been proven that miR-29a has an antifibrotic effect through down-regulation of TGF-β (42). Therefore, both miR-192 and miR-29a are essential regulators of TGF-β mediated fibrogenesis. According to this evidence, Crn had a crucial effect in regulating miR-192 and miR-29a expression and probably had an essential role in reducing renal fibrosis by regulating TGF-β.

## Conclusion

Accumulation of MGO causes ER stress and damages the renal tubules. In this study, we found the occurrence of ER stress and fibrotic alterations by examining the changes in miR-204, 192, and 29a expression in the kidney tissues. Antioxidant evaluations showed that Crn prevents the progression of oxidative stress by reducing MDA levels and strengthening the antioxidant defense system. Also, Crn improved ER stress in MGO-induced DN by regulating the GSH-GLO1-Nrf2 signaling pathway and ER stress-associated microRNAs. Our results will be helpful to better understand the other molecular mechanisms of Crn treatment against high levels of MGO.

## Authors’ Contributions

AA and VR Designed the experiments; AA, VR, SAM, and LK Performed experiments and collected data; AA and VR Discussed the results and strategy; AA Supervised, directed, and managed the study; AA and VR Approved the final version to be published.  

## Conflicts of Interest

There are no conflicts of interest in this study.
